# Agency Modulates the Lateral and Medial Prefrontal Cortex Responses in Belief-Based Decision Making

**DOI:** 10.1371/journal.pone.0065274

**Published:** 2013-06-06

**Authors:** Gui Xue, Qinghua He, Zhong-Lin Lu, Irwin P. Levin, Qi Dong, Antoine Bechara

**Affiliations:** 1 National Key Laboratory of Cognitive Neuroscience and Learning, Beijing Normal University, Beijing, China; 2 Department of Psychology, University of Southern California, Los Angeles, California, United States of America; 3 Brain and Creativity Institute, University of Southern California, Los Angeles, California, United States of America; 4 Center for Cognitive and Behavioral Brain Imaging and Department of Psychology, The Ohio State University, Columbus, Ohio, United States of America; 5 Department of Psychology, University of Iowa, Iowa City, Iowa, United States of America; Heidelberg University, Germany

## Abstract

Many real-life decisions in complex and changing environments are guided by the decision maker’s beliefs, such as her perceived control over decision outcomes (i.e., agency), leading to phenomena like the “illusion of control”. However, the neural mechanisms underlying the “agency” effect on belief-based decisions are not well understood. Using functional imaging and a card guessing game, we revealed that the agency manipulation (i.e., either asking the subjects (SG) or the computer (CG) to guess the location of the winning card) not only affected the size of subjects’ bets, but also their “world model” regarding the outcome dependency. Functional imaging results revealed that the decision-related activation in the lateral and medial prefrontal cortex (PFC) was significantly modulated by agency and previous outcome. Specifically, these PFC regions showed stronger activation when subjects made decisions after losses than after wins under the CG condition, but the pattern was reversed under the SG condition. Furthermore, subjects with high external attribution of negative events were more affected by agency at the behavioral and neural levels. These results suggest that the prefrontal decision-making system can be modulated by abstract beliefs, and are thus vulnerable to factors such as false agency and attribution.

## Introduction

The ability to effectively predict which actions lead to future reward in complex and ever-changing environments is critical for human survival. In addition to a model-free, reinforcement-learning (RL) mechanism that makes predictions by integrating prior reward history via trial and error [1], a model-based mechanism is sometimes useful in forming predictions by implementing knowledge/beliefs of the environment formed through previous experiences [2]. The use of the model-based mechanism has been found under complex and uncertain situations, such as when the reward contingency is either not in existence (e.g., at chance; Xue et al., 2012), or nontransparent (e.g., a tree-search structure; [2], or unstable (e.g., during reversal learning; Hampton et al., 2006), or during social interactions [3].

For example, during decision making involving random events, people consistently make predictions based on false world models, such as the gambler’s fallacy and the hot hand fallacy [4]. The exact pattern is determined by the subjective belief regarding the randomness of the underlying generating mechanisms, such that human performance is generally perceived as showing positive recency (the hot hand fallacy), whereas outcomes generated by natural events are perceived as showing negative recency (the gambler’s fallacy) [5], [6], [7], [8]. Consistently, changes in agency that are responsible for the outcome often lead to different predictions based on the same previous outcomes [5].

The agency manipulation (self vs. non-self generated predictions) can also affect motivation and probability judgments in stochastic decisions, with self-generated predictions creating a phenomenon called the “illusion of control” [9]. At the behavioral level, the “illusion of control” has been consistently found to lead to elevated risk-taking in gambling [9], [10], [11], [12]. At the neural level, the illusion of control leads to stronger activation in the dorsal striatum [13] and the medial prefrontal cortex during outcome processing [12], [14], [15]. Nevertheless, the agency effect on neural responses during decision making has largely been unexplored [16].

Two brain regions, namely the lateral and medial prefrontal cortex, may be subject to modulation by this belief-based decision making. Unlike the model-free, reinforcement-learning mechanism that is based on the prediction error and conveyed via the midbrain and the striatum [17], cumulative evidence has suggested that the prefrontal cortex is primarily responsible for the model- or belief-based decision making [2]. In particular, the medial prefrontal cortex (MPFC) is involved in encoding the values of actions based on the recent response history and outcomes [14], and updating the values under uncertainty [18]. Unlike the striatum during reinforcement learning [17], the value updating in the MPFC is more flexible and is sensitive to the volatility of reward history [19], is contingent upon the state [20] and the context [12] of decision making, and is guided by the belief about the other agent during social interaction [3].

The lateral prefrontal cortex (LPFC), on the other hand, has been posited to play a role in the implementation of model-based decision making, in particular when self-control is required [21]. The LPFC is involved in detecting patterns [22], updating abstract states [23], adapting specific strategies according to the context and state changes [24], and implementing decisions predicted by the world model [21], [25]. Transcranial direct current stimulation of the LPFC changes the use of probability matching and gambler’s fallacy strategies during stochastic decision making [21], [26].

In the present study we aimed at examining how decision-related processes are affected by agency and prior outcomes, two factors that often interact with each other [12]. To this end, we developed a Card Guessing Game where subjects were asked to place a bet on each trial that their pre-specified card would be selected from the two alternatives. They would win the amount they bet if the pre-specified card was selected, but would otherwise lose the same amount. Agency was manipulated by either allowing the subjects to guess which side the winning card was on (i.e., Subjects guess, SG), or letting the computer make the guess (i.e., Computer guess, CG). Unbeknownst to the subjects, the results were predetermined and the winning and losing streaks were systematically manipulated, allowing us to match the outcomes between the two conditions and also to examine the effect of outcome and its interaction with agency on subsequent decisions. We predicted that subjects would bet more under the SG condition than under the CG condition, in line with the “illusion of control” [9]. Moreover, subjects would bet more after a series of losses under the CG condition, a pattern indicative of the gambler’s fallacy [7], [27], whereas they would bet less after a series of losses under the SG condition, a pattern of the hot hand fallacy [8]. At the neural level, we hypothesized that the MPFC and LPFC activity during decision making could be modulated by the interaction of agency and outcomes. However, we expect that our manipulation of agency will not affect all decision makers the same. For example, a more pessimistic person may be less likely to believe that the SG condition will lead to fewer errors. We therefore include a measure of attributional style that will allow us to examine this issue.

## Materials and Methods

### Ethics Statement

The protocol of this study was approved by the Institutional Review Board at the University of Southern California. Informed written consent was obtained from each subject before the experiment.

### Subjects

Eighteen healthy young adults (12 females, 22.28 years of age on average, ranging from 18 to 29) participated in this study. All subjects had normal or corrected-to-normal vision, and were free of neurological or psychiatric history. None were classified as pathological gamblers based on the South Oaks gambling screen [28].

### The Card Guessing Game

**Figure 1:**
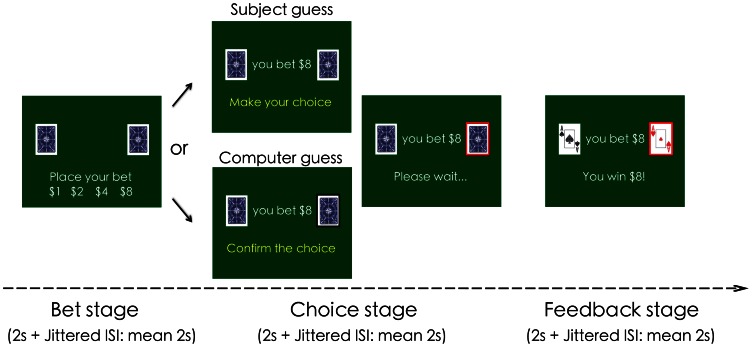
Schema of the card guessing game and experimental design. Each trial consisted of three stages: Bet, Choice and Feedback. During the Bet stage, two folded cards were shown on each side of the screen with one as the winning card, and subjects were asked to place their bet ($1, $2, $4 or $8). After a delay (jittered, mean 2s), the Choice stage started. Under the *Subject guess* (SG) condition, subjects were asked to guess which side their winning card was on. Under the *Computer guess* (CG) condition, the computer made the choice and subjects were asked to simply confirm the computer’s choice. Subjects were told explicitly in advance that the computer made the choice *randomly.* After another delay (jitter, mean 2s), the gamble was revealed and the outcome was displayed for 2 seconds. The next trial started after a delay (jittered, mean 2s). Not shown here, the choices of cards from the last five trials were shown at the top-middle of the screen.

Figure 1A depicts the Card Guessing Game and the experimental design. At the beginning of each session, subjects were asked to choose a card (red or black) as their winning card for the session. Each trial consisted of three stages: Betting, Choice and Feedback. During the Betting stage, subjects were asked to place their bet ($1, $2, $4 or $8). After a delay (mean 2s, ranging from 0 to 4 s), subjects were asked to make a choice. Under the SG condition, subjects were asked to guess which side their winning card was on by pressing one of the two buttons. Under the CG condition, the computer made the guess and subjects were asked to simply confirm the computer’s choice. Subjects were told explicitly in advance that the computer made the guess *randomly.* They were therefore expected to perceive greater control over outcomes in the SG condition than in the CG condition. After another delay (mean 2s, ranging from 0 to 4 seconds), the result was revealed and feedback was delivered for 2 seconds. The next trial started after a jittered delay (mean 2s). There were 63 trials in each 13-minute run. Each subject finished 4 sessions of the Card Game, two under each of the SG and CG conditions, with counterbalanced order across subjects. We did not include the two conditions in the same session because this would interfere with our winning/losing streak manipulation (see below). In order to avoid the wealth effect, they were told in advance that their final payoff would be randomly chosen (by flipping a coin) from one of the four runs.

Unbeknownst to the subjects, for both conditions, the outcome was predetermined using a canonical random sequence generated by a Bernoulli process [21]. The sequence was characterized by (i) equal numbers of black and red cards, (ii) equal probability of card switching, and (iii) exponentially distributed streak lengths. This procedure guarantees that at any streak length, the probability that a winning or a losing streak will continue or break is 50%. To reduce subjects’ memory load, the last five outcomes were presented at the top of the screen. It should be noted that previous literature suggests that on binary choice tasks, one can distinguish sequences of outcomes (e.g. red cards in this task) from feedback sequences (e.g. a winning streak) [5], [27]. The definition of the Gambler's fallacy and “Hot outcome” refers to negative and positive recency of outcomes, respectively, and an increase in bet size after a losing streak would be called the 'stock of luck' belief, which is an opposite bias to the “Hot hand fallacy” [27]. In the present study, this distinction was not necessary as the feedback (e.g., win) was locked to the outcome (e.g., red card), and the gambler’s fallacy and hot hand fallacy thus respectively refer to the negative and positive recency of outcomes.

### Functional Imaging Procedure

Subjects lay supine on the scanner bed, and viewed visual stimuli back-projected onto a screen through a mirror attached to the head coil. Foam pads were used to minimize head motion. Stimulus presentation and timing of all stimuli and response events were achieved using Matlab (Mathworks) and Psychtoolbox (www.psychtoolbox.org) on an IBM-compatible PC. Participants’ responses were collected online using an MRI-compatible button box. Event-related design was used in this fMRI study. An in-house program was used to optimize the design to make sure we could effectively separate the neural responses associated with each stage of the decision making process [29].

fMRI imaging was conducted in a 3T Siemens MAGNETOM Tim/Trio scanner in the Dana and David Dornsife Cognitive Neuroscience Imaging Center at the University of Southern California. Functional scanning used a z-shim gradient echo EPI sequence with PACE (prospective acquisition correction). This specific sequence is designed to reduce signal loss in the prefrontal and orbitofrontal areas. The PACE option can help reduce the impact of head motion during data acquisition. The parameters are: TR = 2000 ms; TE = 25 ms; flip angle = 90°; 64×64 matrix size with resolution 3×3 mm^2^. Thirty-one 3.5-mm axial slices were used to cover the whole cerebrum and most of the cerebellum with no gap. The slices were tilted about 30 degrees clockwise from the AC–PC plane to obtain better signals in the orbitofrontal cortex. The anatomical T1-weighted structural scan was acquired using an MPRAGE sequence (TI = 800 ms; TR = 2530 ms; TE = 3.1 ms; flip angle 10; 208 sagittal slices; 256×256 matrix size with spatial resolution as 1×1×1 mm^3^).

### Attributional Style Measurement

After the fMRI scan, subjects finished the Expanded Attributional Style Questionnaire [30]. The EASQ consists of 24 hypothetical negative life events. Participants were asked to first write down the one major cause of a given event, in an open-ended format, and then rate the cause on a 1- to 7-point scale separately for degree of internality, stability, and globality. The present study focused on the internality vs. externality dimension, and subjects with high internal attribution in this questionnaire were considered displaying a pessimistic explanatory style.

### fMRI Data Preprocessing and Statistical Analysis

Image preprocessing and statistical analysis were carried out using FEAT (FMRI Expert Analysis Tool) version 5.98, part of the FSL package (FMRIB software library, version 4.1, www.fmrib.ox.ac.uk/fsl). The first four volumes before the task were automatically discarded by the scanner to allow for T1 equilibrium. The remaining images were then realigned to compensate for small residual head movements that were not captured by the PACE sequence [31]. Translational movement parameters never exceeded 1 voxel in any direction for any subject or session. All images were de-noised using MELODIC independent components analysis within FSL [32]. The data were filtered in the temporal domain using a non-linear high pass filter with a 100-s cut-off, and spatially smoothed using a 5-mm full-width-half-maximum (FWHM) Gaussian kernel. A three-step registration procedure was used whereby EPI images were first registered to the matched-bandwidth high-resolution scan, then to the MPRAGE structural image, and finally into standard (MNI) space, using affine transformations [31]. Registration from MPRAGE structural image to standard space was further refined using FNIRT nonlinear registration [33]. Statistical analyses were performed in the native image space, with the statistical maps normalized to the standard space prior to higher-level analysis.

The data were modeled at the first level using a general linear model within FSL’s FILM module. The primary goal was to examine how Bet-related response was modulated by agency and reward history. A full factorial design was used, which included the following two factors: Previous outcome (Win vs. Lose) and Outcome streak (Short (1) vs. Long (> = 2)). It should be noted that due to the nature of our design, there were very few really long streak (streak > = 4) trials. As we expected a monotonic effect of streak length, the streak was categorized this way to make sure we have a sufficient number of trials in each category to achieve more reliable results. All the choices were modeled as a single regressor to enable the comparison between the SG and CG conditions. For the feedback stage, the outcome (gain or loss) was modeled parametrically, separately for short and long streaks. This parametric model accounted for the differences in absolute amount of winning vs. losing caused by subjects’ choice of bet size, thus allowing examination of the effect of agency and streak length on outcome processing. The event onsets were convolved with a canonical hemodynamic response function (HRF, double-gamma) to generate the regressors used in the GLM. Temporal derivatives were included as covariates of no interest to improve statistical sensitivity. The derivative parameters were discarded for the subsequent higher-level analysis.

A higher-level analysis (across sessions within each individual) was used to examine the effect of agency (SG vs. CG), using a fixed-effect model. These were then input into a random-effect model for group analysis using an ordinary least squares (OLS) simple mixed effect model with automatic outlier detection [34]. Unless otherwise stated, group images were thresholded using cluster detection statistics, with a height threshold of *z*>2.3 and a cluster probability of *P*<0.05, corrected for whole-brain multiple comparisons using Gaussian Random Field Theory (GRFT) [35].

We further correlated the neural activities with individual differences in attributional style across participants. Voxelwise correlation between the internality score and neural responses was conducted across subjects. An uncorrected threshold of p<.001 was used for this analysis; thus the result should be considered exploratory.

### Region-of-interest (ROI) Analyses

ROIs were created by drawing a 6 mm sphere around the local maxima of the activations. The left and right caudate and nucleus accumbens (NAcc) were anatomically defined according to the Oxford-Harvard Probability map (using 0.25 probability threshold) included in the FSL package, to examine possible subtle effects of agency on reward processing. Using these regions of interest, ROI analyses were performed by extracting the parameter estimates (betas) of each event type from the fitted model and averaging across all voxels in the cluster for each subject. Percent signal changes were calculated using the following formula: [contrast image/(mean of run)] × ppheight × 100%, where ppheight is the peak height of the hemodynamic response versus the baseline level of activity [36]. Correlations between behavioral and ROI data were defined by Pearson product–moment correlations.

## Results

### Behavioral Results: Subjects’ Bets were Modulated by Agency and Reward History

We examined whether subjects’ bet size was modulated by previous outcome (win vs. lose), streak length (1–5 representing number of consecutive winning or losing outcomes) and agency (SG vs. CG). Within-subject ANOVA revealed no significant three-way interaction (F(4,68) = 1.88, p = 0.12), but significant interactions between outcome and agency (F(1,17) = 4.46, p = .05), as well as between outcome and streak length (F(4,68) = 2.91, p = 0.03). Further analysis suggests that under the SG condition, subjects’ bet size was not affected by previous outcome (F (1, 17) = 1.02, p = .40), streak length (F(4,68) = 0.20, p = .66) or their interaction (F (4, 68) = 1.50, p = .21). By contrast, there was a significant streak by outcome interaction in the CG condition (F(4, 68) = 3.08, p = .02) whereby subjects increased their bet size as the losses continued (F(4,68) = 6.27, p = .0002), but did not change their bet as wins continued (F(4,68) = .046, p = .76).

**Figure 2:**
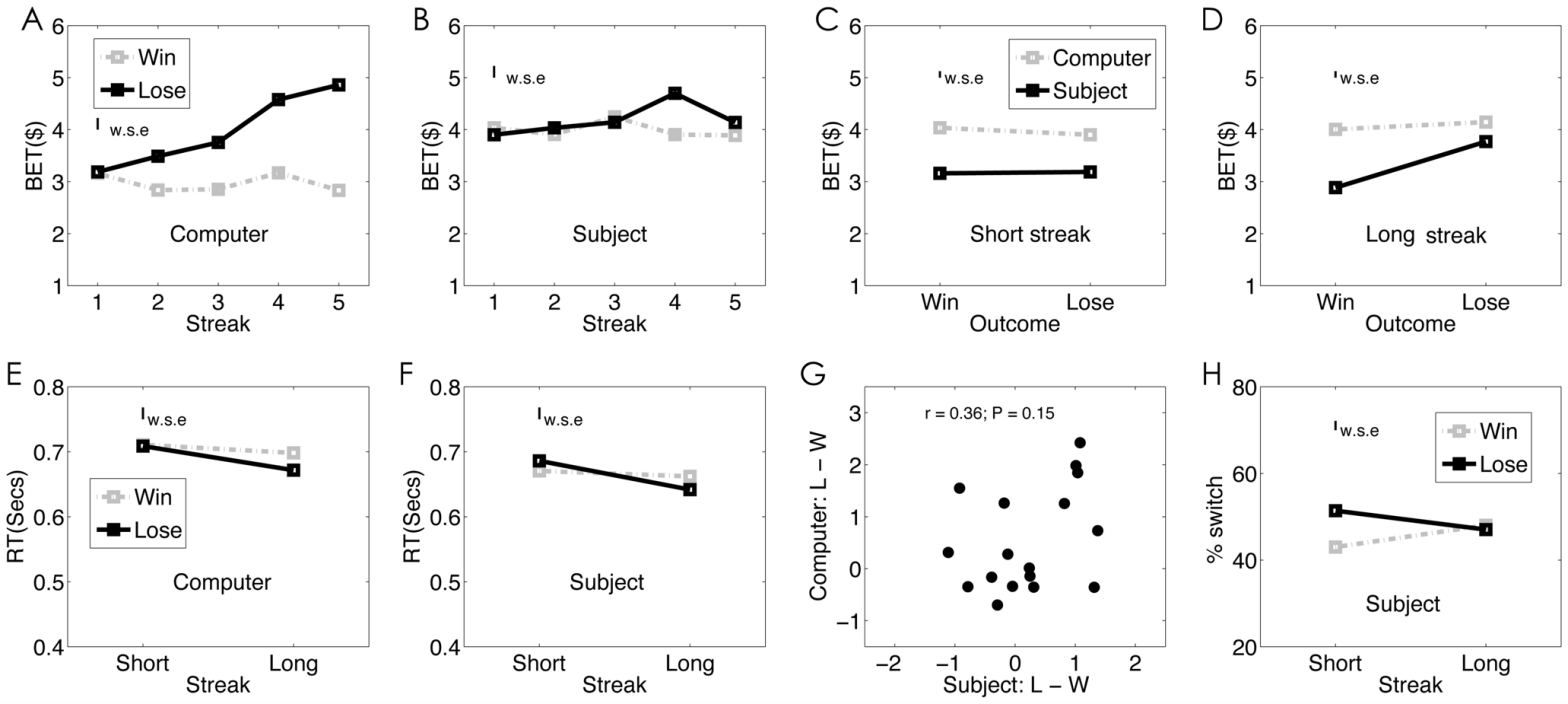
Behavioral results. The averaged bet size was plotted as a function of previous outcome (win vs. loss) and streak length (1 to 5), separately for the CG (A) and SG (B) conditions. The same result was plotted again by separating the streak into short (1) and long (> = 2) streaks, which clearly showed the outcome by agency interaction in the long streak (D), but not the short streak (C) condition. E & F: Reaction time as a function of previous outcome and streak length (short vs. long). (G) The correlation of the gambler’s fallacy effect (as measured by the difference between bet size after loss(es) than after win(s)) between the CG and SG conditions. (H) The switch pattern of subjects’ choices under the SG condition. The small bar on the top left of each plot indicates the within-subject error (w.s.e).

The outcome by agency interaction was more clearly demonstrated by separating the results for short and long streaks (Figure 2 C & D). Under the short streak condition, there was a main effect of agency (F(1,17) = 17.52, p = .0006) with larger bet sizes in the SG condition, but no outcome by agency interaction (F(1,17) = .67, p = .42). Under the long streak condition, the difference between SG and CG was only significant when subjects won (F(1,17) = 13.12, p = .002), but not when they lost (p (1,17) = 2.24, p = .15); the outcome by agency interaction was marginally significant (F(1,17) = 3.50, p = .079). Analysis of response times revealed no significant main effect or interaction (ps>.12) (Figure 2 E & F).

Furthermore, the use of the gambler’s fallacy strategy under the CG and SG conditions did not correlate with each other (Figure 2G), providing further evidence that our manipulation had effectively changed subjects’ beliefs about outcome dependency under the two conditions.

We also looked at subjects’ switch patterns in the SG condition, separately for short and long streaks (Figure 2H). There was a significant outcome by streak interaction (F(1,17) = 4.85, p = .04), indicating that subjects showed a trend toward win-stay-lose-shift after short streaks, but no such pattern after long streaks.

In summary, these results show a clear pattern of the gamblers’ fallacy under the CG condition, but not under the SG condition, suggesting that our manipulation effectively altered subjects’ beliefs about outcome dependency.

### fMRI Results: Bet-related Activation was Modulated by Agency and Reward History

**Figure 3:**
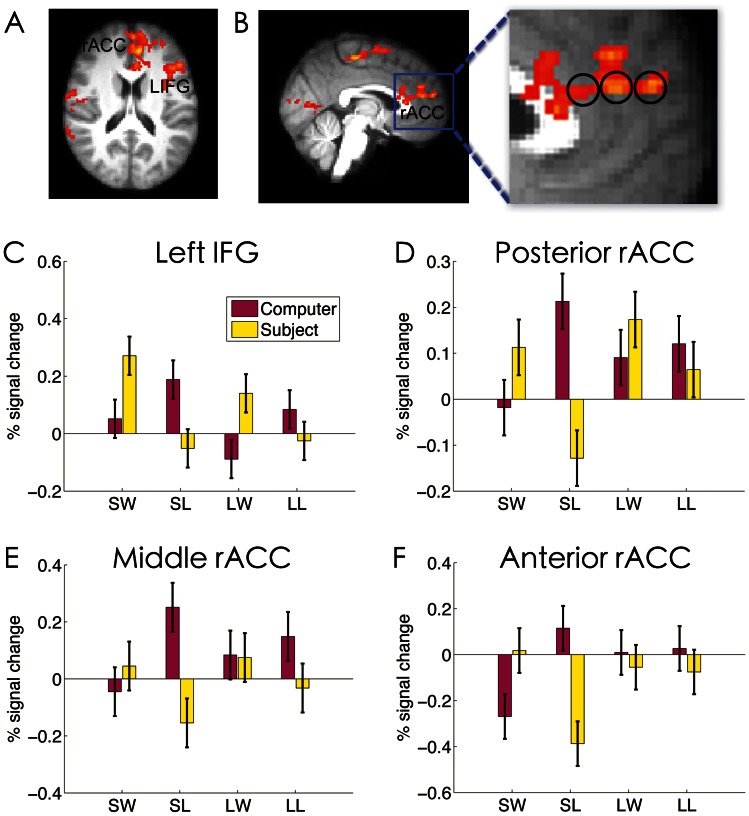
The effect of agency and outcome on bet-related neural activations. Significant agency by outcome interactions in the medial and lateral prefrontal cortex are overlaid on the (A) axial and (B) sagittal slices of the group mean structural images. All activations were thresholded by using cluster detection statistics, with a height threshold of z>2.3 and a cluster probability of P<0.05, corrected for whole-brain multiple comparisons. The top right panel shows the enlarged view of the medial prefrontal cortex cluster, which was further divided into three, i.e., posterior (y = 26), middle (y = 40) and anterior (y = 54) ROIs to examine the possible functional dissociations. To further probe the interactions, the middle and bottom panel show the plots of percentage signal change in (C) the left IFG, (D) the posterior, (E) middle, and (F) anterior rACC, as a function of agency, outcome and streak length. Error bars denote within-subject error. SW: short-win; SL: short-loss; LW: long-win; LL: long-loss.

During the Bet stage, there were significant interactions between agency and previous outcome in the left inferior frontal gyrus (LIFG, x/y/z values in the Montreal Neurological Institute (MNI) coordinate system of −42, 22, and 18; Z = 3.36), and the rostral anterior cingulated cortex (rACC, x/y/z: −6,40,18; Z = 3.65) (Figure 3 A & B). Other regions showing the same task by streak interaction include the left (x/y/z: −62, −14, −20, Z = 3.83) and right (x/y/z: 54, −28, −6, Z = 3.80) middle temporal gyrus, the left lingual/fusiform gyrus (x/y/z: −26, −56, −6, Z = 3.84), and the right lingual gyrus (x/y/z: 12, −68, −4, Z = 3.69).

To further probe the interaction, ROI analysis indicated that for the LIFG, there was a stronger activation when making decisions after wins than after losses in the SG condition (F(1,17) = 7.09, p = .016), but a reversed pattern in the CG condition (F(1,17) = 4.13, p = .058) (Figure 3C). Although the overall response was weaker under long streaks than under short streaks (F(1,17) = 6.08, p = .025), the pattern of interaction holds for both short and long streaks (ps = .013), as indicated by the absence of a significant 3-way interaction between previous outcome, agency and streak length (F(1,17) = 0,41, p = .53).

For the rACC (Figure 3E), there was a trend towards stronger activation when making decisions after wins than after losses under the SG condition (F(1,17) = 2.98, p = .10), whereas under the CG condition there was stronger activation for decisions made after losses than after wins (F(1,17) = 5.63, p = .03). Although the interaction tended to be stronger under short streaks (F(1,17) = 9.08, p = .008) than under long streaks (F(1,17) = 1.87, p = .19), the three-way interaction was not significant (F(1,17) = 1.79, p = .19).

As the rACC/MPFC region is relatively large and previous study suggested functional dissociations within this area [37], we did a further exploratory analysis to examine the response pattern in the subregions. We thus defined two additional equal sized ROIs, one anterior (y = 54) and one posterior (y = 26) to the middle rACC (y = 40). Very similar patterns were found in these two subregions as in the middle rACC (Figure 3D–F).

**Figure 4:**
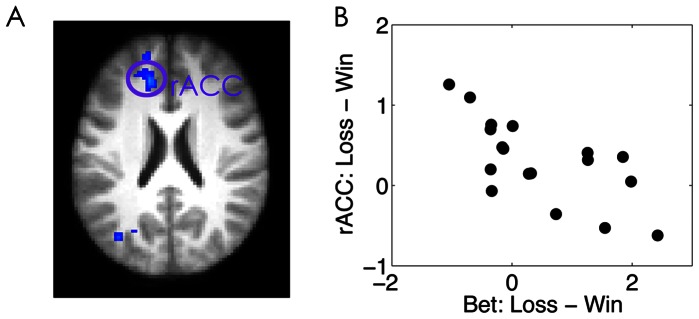
Cross-subject correlation results. (A) The rACC that showed negative correlation between bet size changes and neural activation change as a function of prior outcome was overlaid on an axial slice of the group mean structural image. For display purposes, the activation map was shown at Z>2.3. (B) Scatter plot of the correlation. Please note the scatter plot is only used to check possible outliers. The correlation coefficient should be treated cautiously due to the double-dipping issue.

We further correlated the bet size change as a function of prior outcome with the corresponding neural changes in the rACC. We found for the CG condition where the bet size was significantly modulated by prior outcome, subjects who showed greater neural activation increases during decisions in the rACC (x/y/z: −14,36, 22, Z = 3.60) showed smaller increases in bet size (Figure 4), consistent with a previous observation that the rACC conveys a warning signal to reduce risk taking [38].

### How Decision and Brain Response were Affected by Attribution Style

**Figure 5:**
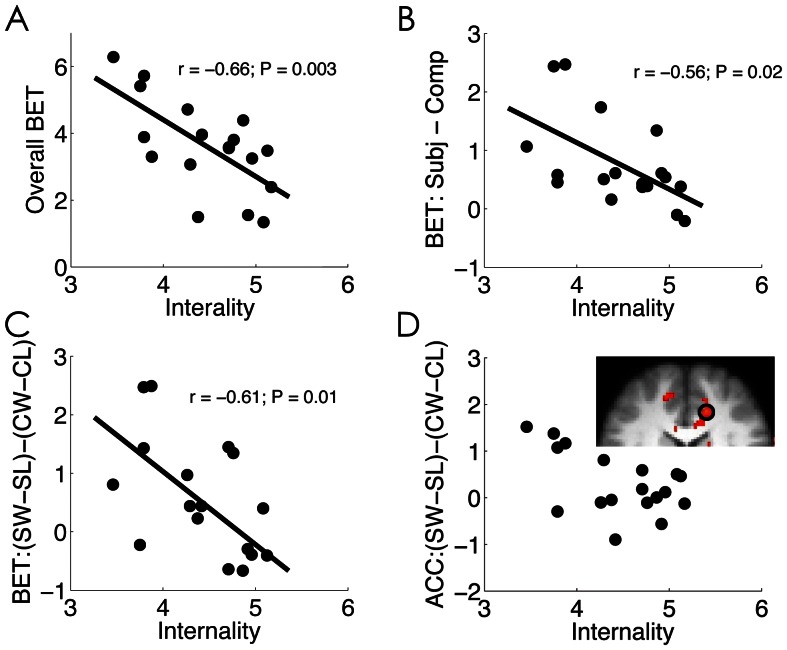
The effect of attributional style on decision making. Scatter plot of the internal attributional style and the (A) overall bet size, (B) behavioral modulation of agency on bet size, (C) behavioral modulation of agency and outcome on bet size, and (D) neural modulation of agency and outcome on rACC activation. Negative correlation between neural modulation and internality in the rACC was overlaid on an axial slice of the group mean structural image. For display purposes, the activation map was shown at (Z>2.3). Please note that the scatter plot in panel D is only used to check possible outliers. The correlation coefficient should be treated cautiously due to the double-dipping issue. SW: subject win; SL: subject loss; CW: computer win; CL: computer loss.

Next, we examined the role of attribution style on subjects’ decision making (Figure 5). We found that the participants with weaker internal attribution of negative events (i.e., less pessimistic) on average bet more than those with stronger internal attribution style (r = −0.66, p = .003) (Figure 5A); they also showed more increase in their bet size under the SG condition as compared to the CG condition (r = −0.56, p = .02) (Figure 5B). In addition, their average bet size was modulated more strongly by the interaction of outcome and agency (r = −.61, p = .01) (Figure 5C). Consistently, their neural activity in the rACC (x/y/z: −14, 38,14, Z = 3.13) was also more strongly modulated by the interaction of outcome and agency (Figure 5D).

### Neural Differences between Active and Passive Choices

**Figure 6:**
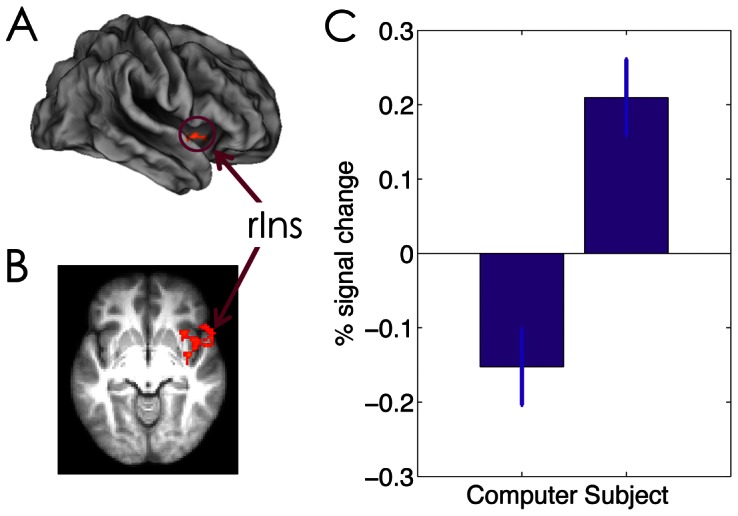
The effect of agency on choice-related activation. Stronger activation in the right insula is rendered onto a population-averaged surface atlas using multi-fiducial mapping [77], and is overlaid on the (B) axial slices of the group mean structural image. Activations were thresholded using cluster detection statistics, with a height threshold of z>2.3 and a cluster probability of P<0.05, corrected for whole-brain multiple comparisons. (C) Bar graph of the percentage signal change in the right insula during choice stage as a function of agency. Error bars denote within-subject error.

We then compared the neural activation between the SG and CG conditions during the choice stage. Consistent with previous observations [16], [39], we found that internal agency (SG>CG) was associated with stronger activation in the right insular cortex (x/y/z: 38, −4, −6, Z = 3.68) (Figure 6).

### The Effect of Agency and Reward History on Outcome Processing

**Figure 7:**
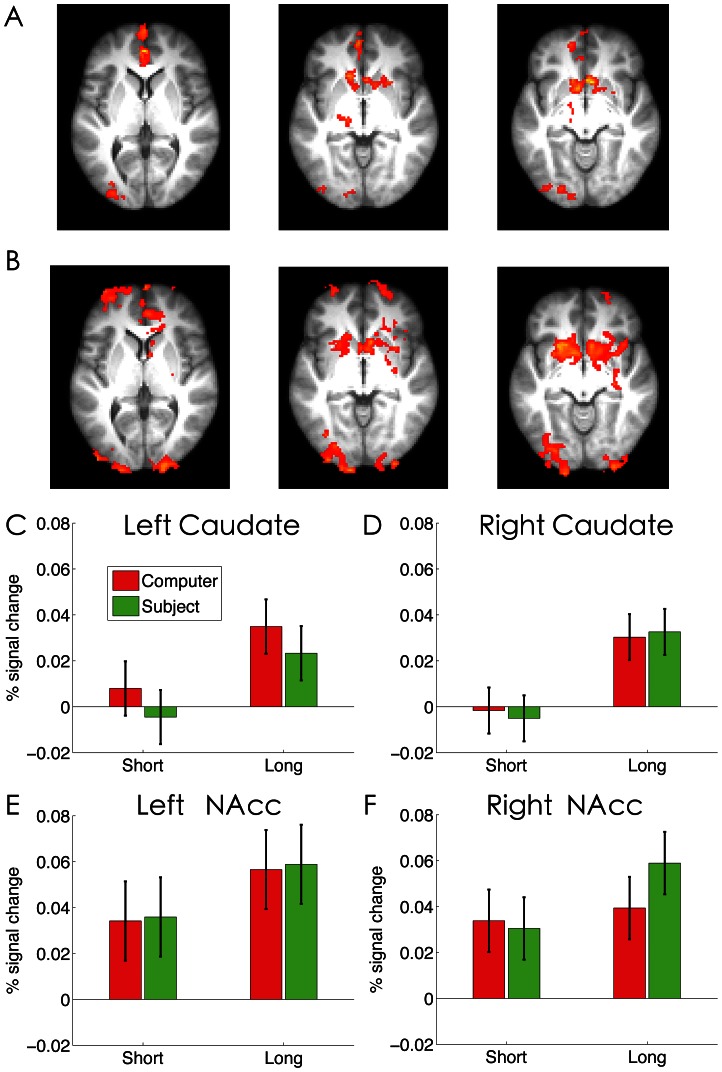
The effect of agency and streak on feedback-related activations. Significant positive parametric modulation by experienced reward is overlaid on the axial slices of the group mean structural image, separately for the (A) CG and (B) SG conditions. Activations were thresholded by using cluster detection statistics, with a height threshold of z>2.3 and a cluster probability of P<0.05, corrected for whole-brain multiple comparisons. Direct comparison revealed no significant effect of agency. Bar graphs show the percentage signal change, as a function of streak length and agency, in the anatomically defined (C) left and (D) right caudate, and (E) left and (F) right nucleus accumbens (NAcc). Error bars denote within-subject error.

Finally, we examined how agency and reward history (winning or losing streak) modulated outcome processing. Because subjects’ bets were modulated by agency, previous outcome and streak length, a parametric analysis was used to examine how the brain response was modulated by the magnitude of the experienced outcome (losses coded as negative and wins coded as positive), separately for short and long streaks. As we found no significant differences between short and long streaks at the whole-brain corrected level of significance, the averaged response across both short and long streak was calculated (Figure 7A & B). Consistent with many previous observations [38], [40], strong positive modulation by experienced outcome was found in the ventral medial prefrontal cortex (computer: x/y/z: −2, 44, 2, Z = 4.07; Subject: x/y/z: −4, 38,10, Z = 3.05), the left (computer: x/y/z: −8,10, −8, Z = 3.60; Subject: x/y/z: −10, 8, −12, Z = 3.95) and the right NAcc (computer: x/y/z: 6, 16, −8, Z = 4.03; subjects: x/y/z: 8, 10, −12, Z = 3.55), but no effect of agency or streak was significant at the whole-brain correction level.

To probe the possible subtle agency effect in the striatum [13], we conducted an unbiased ROI analysis by extracting the BOLD signal within the anatomically defined caudate and NAcc. Even with this more sensitive ROI approach, we still found no significant effect of agency in the caudate (ps>.19) (Figure 7C & D) or in the NAcc (ps>.72) (Figure 7 E & F). Instead, there was stronger activation under long streaks than under short streaks in the left (F(1,17) = 5.51, p = .03 ) and right caudate (F(1,17) = 9.26, p = .007) (Figure 7C & D). Only a marginal effect of streak was found in the left NAcc (p = .07) but not in the right NAcc (p = .14) (Figure 7E & F).

## Discussion

The present study examined the cognitive and neural processes engaged in belief-based decision making involving random events, how the behavioral data and neural activity were modulated by agency (the belief that one is in control of the decision outcome), and how they were also influenced by a particular personality trait, attributional style. We found that both agency and attributional style significantly affected value updating from previous outcomes and subsequent decisions, and also modulated the activations in the medial and lateral prefrontal cortex, two important regions involved in value-guided decision making. These results provide novel evidence that human decision making mechanisms are dynamically and flexibly shaped by our cognitive model of the environment, which is deeply rooted in our experiences and personality, even when such reliance on the world model is proven to be false and maladaptive.

Consistent with several previous behavioral observations [9], [10], [11], [12], our healthy subjects also showed a strong pattern of agency (or “illusion of control”) in the card guessing game, as they bet significantly more under the SG condition than under the CG condition, especially under short streaks and long winning streaks, even though wins and losses were governed purely by chance. Unlike the study by Clark et al. [12] that explicitly probed the subjects’ conscious intention (using verbal reports) to continue gambling, the present study used bet size as a subjective index of their tendency toward risk-taking, which is arguably a more direct and sensitive measure that tapped more into their unconscious decision making processes guided by subjective beliefs or “world models”[41]. Still, this approach would not increase their conscious knowledge about the task structure and thus there should be less contamination of reflective processes [42]. In the present study, the outcomes were predetermined and strictly matched across two conditions, thus any difference in bet size could not be attributed to differences in outcomes, but instead reflect the illusion of control.

In addition to the increased level of motivation and risk-taking, as reflected by the increased bet size, the agency manipulation has also been shown to affect the world model regarding the outcome dependency, such that successive outcomes generated by computer were perceived as negatively correlated [5], [6], thus promoting the gambler’s fallacy strategy characterized by increasing bet size after a series of losses. There was a slight trend of decreases in bet size after a series of wins, but this was not significant. No such pattern was displayed in the SG condition. Consistent with a previous filed study on casino gamblers that only revealed a small hint of the hot hand fallacy in the Roulette game [27], our healthy college students who are not pathological gamblers on average did not show the hot hand fallacy under the SG condition. Across subjects, the use of the gambler’s fallacy strategy under the CG and SG conditions did not correlate with each other, providing further evidence that our manipulation had effectively changed subjects’ beliefs about outcome dependency under the two conditions.

At the neural level, it is striking that the lateral and medial prefrontal cortex responses were reversed by the subtle manipulation of agency (SG vs. CG), even when everything else was kept constant. Notably, the observed interaction between outcome by agency in the lateral and medial prefrontal cortex did not simply mirror the bet size or reaction time, thus excluding the possibility that they merely reflect decision risk or cognitive demand. Still, unlike previous studies [16], the decision requirement at the Bet stage under both conditions was identical, i.e., actively choose the bet size. These results thus provide compelling evidence to support the role of agency in modulating the adaptive decision making network.

The medial prefrontal cortex is not only involved in monitoring errors or simply reinforcement learning, but also in integrating reward history according to the changing contextual and abstract state of information [18], [43], such as the votalility/salience of the reward [19], the history of actions and outcomes [44], and the abstract high-order state of reward contingency [20]. During social interaction, the medial prefrontal cortex is important for understanding the intentions of other individuals [45]. Accordingly, it has been implicated in strategic reasoning in complex social games [37], and the representation of belief-based prediction errors [3]. The medial prefrontal cortex thus enables the flexible update of reward history to guide future choices, which is underscored in the present study by demonstrating that belief-based decision making was largely guided by the subjective world model that is shaped by context (e.g., agency) and personal traits (see below).

We found the rACC activation was negatively correlated with individuals’ risk taking [25], [38]. This evidence, although preliminary and exploratory in nature, is nevertheless consistent with previous observations emphasizing its role in affective decision making. Patients with damage in the medial prefrontal cortex, who could not use the affective signal to guide decision making, showed profound decision deficits characterized by the gambler’s fallacy [46] [47], [48], [49]. These patients had explicit knowledge of the good and bad choices [50], and also showed normal switching to other choices after receiving big losses, but just returned to bad choices more quickly [51]. Consistently, rACC lesion monkeys also showed normal switching of their movement when the reward contingency was reversed, but were more likely to revert back to the incorrect, unrewarded movement [44]. Further analysis showed that the influence of past trials on subsequent decisions declined significantly more quickly on rACC lesion monkeys [44], which is in line with the observation on the MPFC patients who showed a stronger recency effect in their decisions [52]. We think the affective nature of the value coded in the MPFC [46], [53] provides a good mechanistic account of this observed recency effect due to MPFC hypofunctioning, and also the dissociation between conscious knowledge and actual decisions. That is, rational knowledge itself is not enough to guide rational decisions, not to mention when the knowledge is wrong. In our case, the affective signal would help to resist the temptation to take more risk as guided by the false world model.

We also found that the agency manipulation significantly modulated the lateral prefrontal cortex (LPFC) activation. Consistent with our hypothesis, there was stronger LPFC activation when making decisions after losses than after gains under the CG condition [25]. Strikingly, under the SG condition where a different belief regarding the outcome dependency was involved, the pattern of LPFC activation was completely reversed, showing stronger activation after gains than after losses. This provides clear evidence to support the role of the LPFC in the implementation of model-based decision making, in particular when self-control is required [21]. Given that the LPFC is an important region for conflict resolution [54] and set switching [55], it is also consistently involved in expressing self-control during inter-temporal choices [56], food choices [57], and interpersonal interactions [58]. A recent developmental study also suggests the anatomical and functional development of LPFC correlates with the use of strategic social behaviors [59]. In the sense that self-control is guided by a world-model or belief that favors long-term goals and social interests instead of immediate and selfish reward, these observations underscore the LPFC’s role in implementing the belief-based decisions. Importantly, as the world-model could be either true or false, the LPFC thus could be involved in both rational and irrational decision-making [21]. From this perspective, it is no surprise that when being “hijacked” by a wrong world model, individuals with strong cognitive capacities, although often making more rational decisions, can be led to make more maladaptive decisions [60]. Anodal transcranial direct current stimulation that enhances the LPFC function has also been shown to promote maladaptive decisions [21].

Our study is among the first to examine the effects of attributional style on risky decisions. Behavioral studies have shown that subjects with internal attribution of negative affect display a pessimistic explanatory style and low self-efficacy [30], which is associated with low motivation and risk-taking [61]. Consistently, we found that subjects with high internal attribution likely attributed losses to themselves, overall bet less, and their bet size was less modulated by agency manipulation. In addition, attributional style affected the beliefs about outcome dependency, and also the activation in the rACC. Notably, the EASQ only measures the attribution of negative events, which might not necessarily correlate with that of positive events. Future studies need to examine how attributional style for positive and negative events differentially interact with agency to affect risky decision making.

We also found a strong effect of agency in the choice stage. Behavioral data suggest that subjects actively adjusted their guesses based on previous outcomes. fMRI results revealed strong activation in the right insula when subjects made choices in the SG condition, which is consistent with the meta-analysis results implicating its role in self agency on motor tasks [39]. However, the insula activation is unlikely caused by the increased match between action and feedback [62], but instead may reflect the strong arousal effect on motivated behavior in the SG condition [16], [63]. This interpretation is consistent with the role of the right insula in translating interoceptive and homeostatic signals into feeling states that energize reward seeking behaviors [64], [65], [66], [67]. Still, because the bet size was larger under the SG condition and thus the stakes were higher, this increase in activation might also reflect the increased risk level [68].

Unlike previous studies [13], we did not find strong modulation in the dorsal striatum by agency during outcome processing. Presumably, our agency manipulation did not completely abolish the “illusive” self-agency under the CG condition as subjects could still actively choose how much to bet. In addition, as the subjective value function is nonlinear [69], our use of the linear value function may have underestimated the neural effect of larger rewards and punishments which could lead to reduced differences between the conditions. The absence of difference in the ventral striatum, however, is consistent with the observation that its activation is not determined by the instrumental demands [17]. We found that bilateral caudate activation was modulated by streak length, such that a winning streak elicited stronger activation in this area than did a single win. A similar trend was found in the bilateral NAcc, consistent with a previous study showing elevated reward signals under long streaks [70].

In sum, cumulative evidence has suggested that decision making involves multiple mechanisms [2], [20], [21], [23], [71], [72]. For decisions under uncertainty where the reward contingency is at chance level, we found strong modulation of agency and attributional style on the medial and lateral prefrontal cortex, which are involved in model-based value updating and decision strategy implementation. Given that human decision making faculties are so vulnerable to wrong subjective beliefs, and that cognitive distortions are commonly observed in people with psychiatric conditions, including depression [73], antisocial behavior [74], and pathological gambling [75], the combination of neurofunctional measures of the decision makers’ cognitive and brain capacities as well as their subjective beliefs might be a fruitful direction in advancing our understanding of their decision making deficits, as well as in determining the moral and legal consequences of their decisions [76].
